# 

**Published:** 1905-11

**Authors:** 


### CONTENTS.

ORIGINAL COMMUNICATIONS—

The Case of Yellow Fever Recently Occurring in Atlanta, Ga. By William B. Summerall, M.D., Atlanta, Ga. .... 505
Belharzia Hematobium; Report of Seven Cases. By Claude A. Smith, M.D., Atlanta, Ga. .... 515
Medical Witness. By D. L. Field, M.D., Jeffersonville, Ind. .... 518
Some Cases of Enuresis. By J. McF. Gaston, A.M., M.D., Atlanta, Ga. .... 521
Epilepsy. By John W. Selman, M.D., Greenfield, Ind. .... 524
Hepatitis. By D. L. Field, M.D., Jeffersonville, Ind. .... 530
A Plea for More Moral Restraint for Students in Our Medical Colleges .... 533

EDITORIAL NOTES AND COMMENTS—

The Atlanta College of Physicians and Surgeons .... 537
The Atlanta School of Medicine .... 540
Demonstration of Spirochæta Pallida .... 542

NEWS AND NOTES .... 544

CONTENTS CONTINUED .... X

